# Pain perception, opioid consumption and mobility following lateral compression pelvic ring injuries: a two-year prospective cohort study

**DOI:** 10.1007/s00590-024-04128-w

**Published:** 2025-01-11

**Authors:** Camryn C. Therrien, Kaj ten Duis, Jean-Paul P. M. de Vries, Inge H. F. Reininga, Frank F. A. IJpma

**Affiliations:** 1 Department of Trauma Surgery, University of Groningen, University Medical Center Groningen, Groningen, The Netherlands; 2 Department of Surgery, University of Groningen, University Medical Center Groningen, Groningen, The Netherlands

**Keywords:** Pelvic ring injury, Lateral compression injury, Pain, Opioid use, Mobility, Walking

## Abstract

**Purpose:**

A prospective longitudinal cohort study was performed to gain insight into the course of recovery in terms of pain, opioid consumption, and mobility in patients with a lateral compression (LC) pelvic injury.

**Methods:**

Adult patients with an LC injury, without any cognitive disorders or limited mobility and who could communicate in Dutch were asked to participate. Pain in terms of NRS (numeric rating scale, range 0–10), opioid use and mobility were recorded at eight time points: at hospital admission, and three days, one week, six weeks, three months, six months, one year and two years after the injury. A sub-analysis was performed for nonoperatively and operatively treated patients.

**Results:**

Ninety-seven patients were included, of which 23 (24%) were treated operatively and 74 (76%) conservatively. Pain at rest and exertion, were highest upon admission (mean NRS of 3.4 (SD = 2.6) and 4.4 (SD = 2.8), respectively) but decreased within six weeks (mean NRS of 0.8 (SD = 1.6) and 2.0 (2.0), respectively). After two years, the mean NRS was 0.5 (SD = 1.6) and 0.9 (SD = 2.1), respectively. Upon admission, 85% were given opioids, however only 11% used opioids after three months and 4% after two years. At three months, 35% were walking using walking aids and 58% were walking independently. After two years, 98% were walking independently.

**Conclusions:**

Pain rapidly decreased within the first six weeks. Most patients did not need opioids after three months. Furthermore, most patients were walking with walking aids after six weeks. After two years, few patients experienced pain, used opioids or had difficulties walking.

**Supplementary Information:**

The online version contains supplementary material available at 10.1007/s00590-024-04128-w.

## Introduction

Traumatic pelvic ring injuries encompass all disruptions occurring in the bony and ligamentous structures of the pelvis, resulting from an accident [1]. The incidence of pelvic ring injuries in the Netherlands is reported to be 14.3 per 100,000 inhabitants per year [1]. These injuries are shown to significantly impact patients’ physical functioning and quality of life [2–7]. Patients with pelvic ring injuries experience a period of pain and impaired mobilization [6, 8], with evidence indicating long-term discomfort and dysfunction [3, 9].

According to the Young and Burgess classification, pelvic ring injuries can be grouped into lateral compression (LC), anterior–posterior compression (APC) or vertical shear injuries [10, 11]. This classification is based on the direction of force during the injury, which is important as it determines specific injury patterns, with injuries in both the anterior and posterior pelvic rings [12]. LC injuries are the most commonly occurring pelvic ring injuries [13]. These injuries often involve fractures of the pubic rami and sacrum on the side of impact, along with an internal rotation of the hemipelvis [11, 14]. Pain and difficulties with mobilization, specifically regarding walking ability, are key factors in the recovery process [5, 6]. Unfortunately, the course of recovery in terms of pain perception, opioid use, and mobility for LC injuries specifically remains unclear. Furthermore, non-operative treatment of LC1 injuries is highly controversial in the literature [6, 15], with no consensus of treatment among trauma surgeons internationally [16]. Prospective data on the course of recovery of nonoperatively treated LC injuries, particularly concerning pain levels, opioid use, and mobility, is currently lacking in the literature. To our knowledge, there are currently no prospective studies that investigate these key factors of the recovery process of LC injuries.

Therefore, a longitudinal prospective study was conducted in a level-1 trauma and referral center for the treatment of pelvic injuries. Longitudinal data regarding pain, pain medication use, and mobility were recorded from the time of admission until the two-year follow-up. This allowed us to gain insight into the patient’s perception of pain, consumption of pain medication and mobility after an LC injury to better understand the course of recovery of this injury. The research question is therefore: What is the course of recovery in terms of pain perception, opioid consumption, and walking ability in patients with an LC injury?

## Patients and methods

### Patients

A prospective longitudinal cohort study was performed over five years. Patients over the age of 18 who were treated, either surgically or conservatively, for a lateral compression pelvic ring injury in the University Medical Center Groningen (UMCG), a level 1 trauma center and referral center for pelvic injuries in the north of the Netherlands, were included.

All patients who did not have any known cognitive disorders, nor a previously deformed pelvis or limited mobility and who were able to communicate in the Dutch language were informed about the study and asked to participate. Patients who were transferred to a different region to complete their follow-up or those with a tertiary referral to the UMCG that were only seen once at the outpatient clinic were excluded. Patients were treated according to standard practice. The choice of treatment often relied on a combination of clinical presentation, imaging, and attempts for mobilization in terms of walking ability, and was a shared decision-making process between the pelvic trauma surgeons and the patient. The local Medical Ethical Review Board reviewed the methods employed and waived further need for approval (METc 2017/543).

### Data collection

Data on the patient’s characteristics were prospectively collected from the patient’s electronic records and were directly entered into the database upon clinical presentation. This included information about the injury, treatment, complications, and mortality. Radiographs were also retrieved from the electronic patient records. Two trauma surgeons with more than 5 years of experience in pelvic ring injury surgery assessed the radiographic images (plain anteroposterior, inlet and outlet radiographs and CT scans) of all patients. Pelvic ring injuries were classified according to the Young-Burgess classification [11].

Pain scores, opioid use and walking ability scores were collected by surgeons or specialized nurses using an electronic template in the patient file at the time of hospital admission, three days, one week, six weeks, three months, six months, one year, and two years after the time of injury, respectively.

### Outcome measures

Pain perception was measured at rest and on exertion on a scale of 0–10 using the numeric rating scale (NRS) [17]. The pain recorded was specific to the pelvic area. Furthermore, an NRS of ≥ 4 at rest was used as an indicator of unacceptable pain [18, 19]. The use of opioids (yes or no) was recorded at each time point. Opioid use was recorded as it is often a key indicator of more severe pain [20]. A binary approach to record opioid use was taken because opioids are prescribed to take as needed, so the exact dosage that was consumed was not always available. Furthermore, the use of non-opioid-analgesics was not recorded due to the potential for underreporting [21]. Walking ability was assessed on a mobility score of 0–7, with 0 indicating that a patient is bedridden and 7 indicating that a patient does not have impairments in walking and does not use walking aids. The level of walking ability at that moment was indicated by the patient during the anamneses. The mobility score was further grouped into three groups: scores 0–1 were considered to indicate immobile, 2–4 were mobile only with the use of a walking aid, and 5–7 were independently mobile. The assessment of walking ability was derived from a question regarding mobility in the Majeed Pelvic Outcome score [22].

### Analysis

The statistical analysis was conducted using SPSS software (version 28, IBM Corp). Descriptive statistics were performed to present patient demographics, injury characteristics, and outcomes at each follow-up moment. For normally distributed data, means and standard deviations were used; while, medians and interquartile ranges (IQR) were used for nonnormally distributed data. To illustrate the recovery progression over time, pain, opioid use and mobility for patients with LC injuries were plotted on a line graph. A sub-analysis was performed for patients who were treated nonoperatively and operatively and presented on line graphs. Differences in patient and injury characteristics between the non-operative and operative groups were compared using an independent t-test for continuous variables (comparing means) and a chi-square test for categorical (binary) variables. The comparison of groups is included in the supplementary data.

## Results

### Study population

**Fig. 1:**
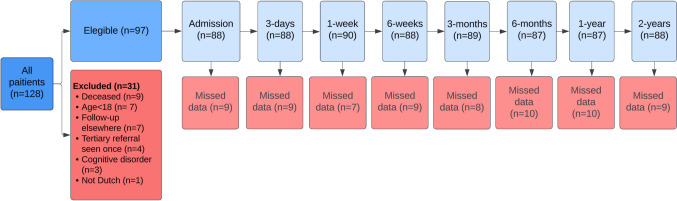
Flowchart of patient involvement

During the five years of the study, 128 patients with lateral compression pelvic injuries were treated in a level 1 trauma center. Of these patients, 97 were eligible to participate, as shown in Fig. 1. Of the patients who were included, the mean age was 55 (SD = 20) and 53% (*n* = 51) were female. Information regarding the injuries and injury mechanisms can be found in Table 1. Regarding treatment, most patients, 74 (76%), were treated conservatively. Conservative treatment consisted of a 6-week limited weight-bearing (20 kg) period with wheelchairs or crutches, followed by weight-bearing as tolerated. Twenty-three patients (24%) underwent operative fixation. Of these, 11 patients (11%) were treated with only anterior pelvic ring fixation (10 plate osteosynthesis and one anterior screw fixation of the pubic rami), 8 patients (8%) with only posterior pelvic ring fixation (three patients with one sacroiliac screw, two patients with two sacroiliac screws, one patient with one trans-sacral screw, one patient with two sacroiliac plates and one sacroiliac screw and one patient with lumbopelvic stabilization), and 4 patients (4%) underwent both anterior and posterior pelvic ring fixation (all four patients had an anterior plate osteosynthesis and three patients had one additional sacroiliac screw and one patient had two additional sacroiliac screws). Furthermore, three patients (3%) were treated with an external fixator. The average time between admission and operation was 4 (SD = 3) days. The length of the hospital admission was an average of 10 (SD = 9) days. Following the admission, 33 (34%) were discharged to a care facility and 64 (66%) were able to go home. The number of patients with missing data at each follow-up moment ranged from 7 to 10, with different patients contributing to the missing data at each time point. These gaps were due to factors such as patients missing their follow-up appointment or surgeons not recording the data.

**Table 1:** Patient characteristics

Patient characteristics	All patients (*n* = 128)	Patients included (*n* = 97)
Female, *n* (%)	67 (52%)	51 (53%)
Age at the time of injury mean (+-SD)	54 (22)	55 (20)
Age < 65, *n* (%)	78 (61%)	56 (57%)
Age > 65, *n* (%)	50 (39%)	41 (42%)
Injury type, *n* (%)		
Lateral compression 1	92 (72%)	71 (73%)
Lateral compression 2	18 (14%)	14 (14%)
Lateral compression 3	18 (14%)	12 (12%)
High-energy trauma*, *n* (%)	86 (67%)	65 (67%)
Isolated pelvic ring injury, *n* (%)	49 (38%)	40 (41%)
Associated lower extremity injuries, *n* (%)	20 (16%)	13 (13%)
Conservative treatment, *n* (%)	111 (79%)	74 (76%)
Operative treatment, *n* (%)	27 (21%)	23 (24%)
Anterior fixation, *n* (%)	13 (10%)	11 (11%)
Posterior fixation, *n* (%)	10 (8%)	8 (8%)
Both anterior and posterior fixation, *n* (%)	4 (3%)	4 (4%)
Deceased, *n* (%)**	10 (8%)	1 (1%)
< 30 days	9 (7%)	0

^*^A high-energy trauma is defined as an assumed impact greater than 20 km/h or a fall from at least twice the patient’s height

^**^Nine patients died from the initial trauma and one from a myocardial infarct

### Pain perception

**Fig. 2:**
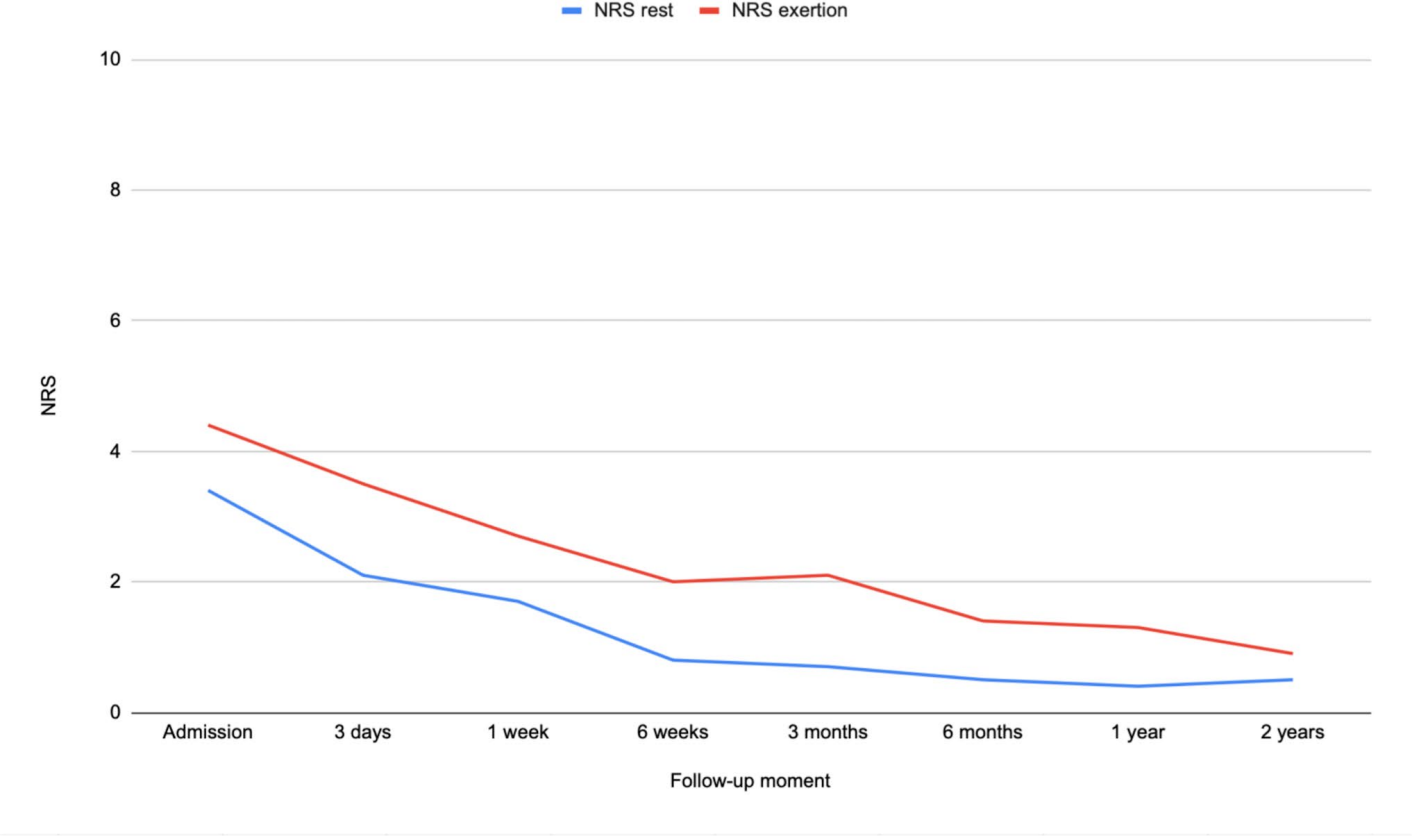
Progression of NRS pain for LC injuries, represented by the mean NRS score at each time point at both rest and on exertion

As visualized in Fig. 2, the mean pelvic-related pain experienced at rest and on exertion decreased over the two years of recovery. The mean NRS scores at each follow-up moment are shown in Table 2. At the time of admission, the mean NRS scores at rest and on exertion were 3.4 (SD = 2.6) and 4.4 (SD = 2.8), respectively. After 6 weeks of recovery, the mean scores decreased to 0.8 (SD = 1.6) and 2.0 (SD = 2.0), respectively. After two years the mean scores were 0.5 (SD = 1.6) on rest and 0.9 (SD = 2.1) on exertion. There were five patients who still experienced unacceptable pain (NRS ≥ 4) at the two-year follow-up. Three of these patients experienced sacral/ lower back pain, one experienced a diffuse pain in the entire pelvis, and one experienced neurological pain originating from the sacral area, radiating into the leg. Two of these patients were treated nonoperatively and three were treated operatively.

**Table 2:** Pain perception, opioid use and mobility score

	NRS rest, mean (SD)	NRS exertion, mean (SD)	Opioids, *n* (%)	Mobility*, mean (SD)
Admission	3.4 (2.6)	4.4 (2.8)	75 (85)	0.3 (0.1)
3 days	2.1 (1.9)	3.5 (2.4)	63 (72)	0.7 (1.1)
1 week	1.7 (1.8)	2.7 (2.0)	55 (61)	1.5 (1.2)
6 weeks	0.8 (1.6)	2.0 (2.0)	20 (23)	3.1 (2.2)
3 months	0.7 (1.5)	2.1 (2.5)	10 (11)	5.2 (2.1)
6 months	0.5 (1.6)	1.4 (2.4)	7 (8)	6.2 (1.8)
1 year	0.4 (1.8)	1.3 (2.7)	3 (3)	6.4 (1.5)
2 years	0.5 (1.6)	0.9 (2.1)	4 (4)	6.8 (0.9)

^*^Walking ability was assessed on a mobility score of 0–7, with 0 indicating that the patient is bedridden and 7 indicating that the patient does not have impairments in walking

### Opioid use

**Fig. 3:**
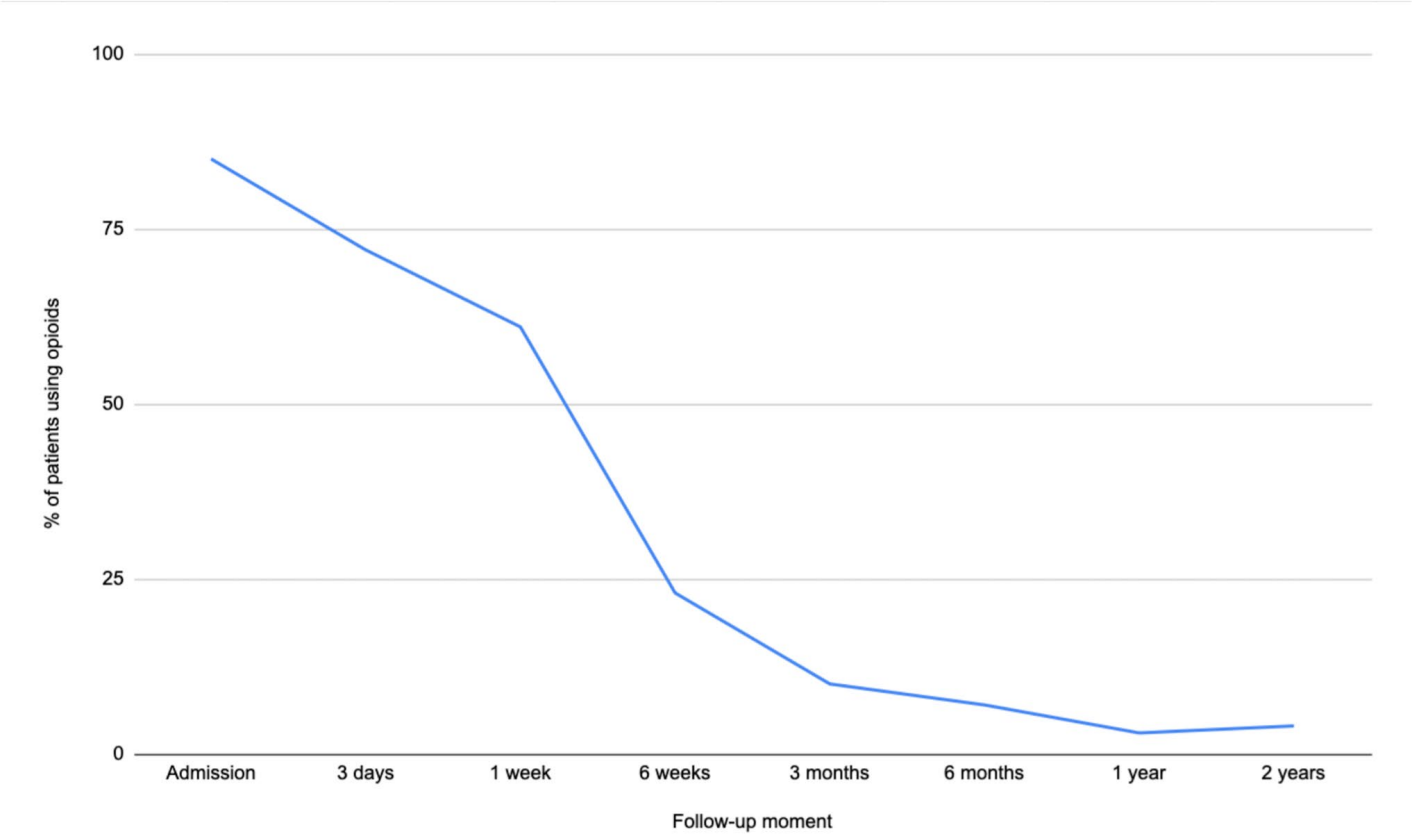
Progression of opioid use for LC injuries, represented by the percentage of patients who use opioids at each time point

Opioid consumption decreased in the first two years of recovery, as demonstrated in Fig. 3. See Table 2 for the number of patients who were prescribed opioids at each time point. During admission, 85% (*n* = 75) of patients were prescribed opioids. However, this decreased substantially to 11% (*n* = 10) after 3 months and further declined to 4% (*n* = 4) after 2 years. Of the four patients still using opioids after two years, one experienced sacral pain with an NRS of 8 at rest and 9 on exertion. Another patient experienced diffused pelvic pain with an NRS of 6 at rest and 7 on exertion. A third patient suffered from lower back/sacral pain, with an NRS of 7 at rest and 9 on exertion. Lastly, one patient experienced groin pain with an NRS of 0 at rest and 3 on exertion. Two of these patients were treated nonoperatively and two were treated operatively.

### Walking ability

**Fig. 4:**
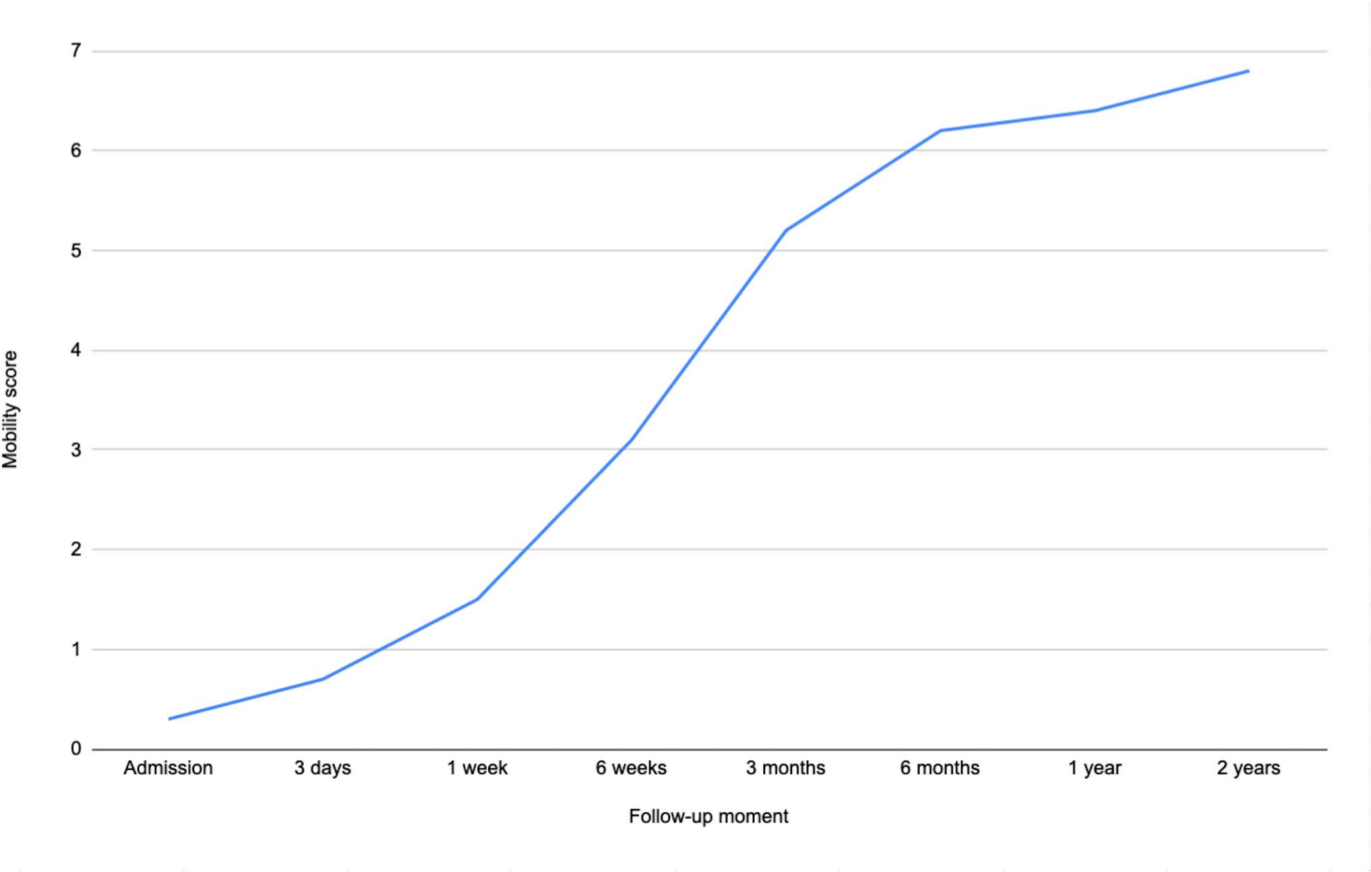
Progression of mobility for LC injuries, represented by the mean mobility score

**Fig. 5:**
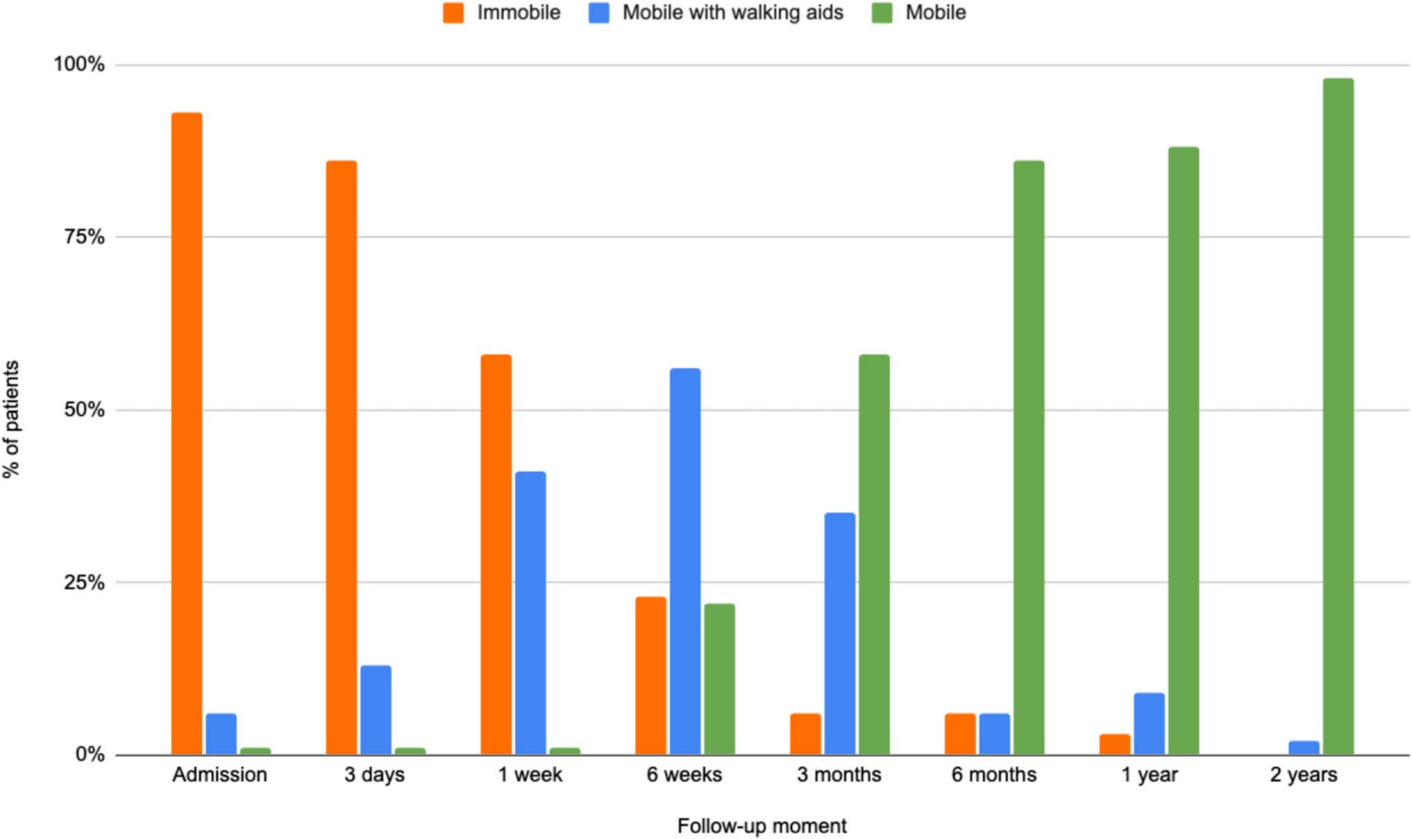
Walking ability of patients at each follow-up moment

The mean mobility scores are presented in Table 2 and Fig. 4. In Fig. 5 it can be seen that the majority of patients were immobile during the first week post-injury. However, at the 6-week follow-up, there was a marked increase in the number of patients who were mobilizing in terms of walking ability, either with the aid of a walking device or independently. After two years, nearly all patients were walking independently. There were two patients who did not, and they both used a rollator walker. One due to pelvic pain experienced in a 65-year-old patient with a body max index (BMI) of 44.8 kg/m^2^ and an 80-year-old patient with coxarthrosis. Of the 2 patients who were unable to walk independently, one was treated nonoperatively and one was treated operatively. Both patients were able to walk independently before the accident.

### Sub analyses for nonoperatively and operatively treated patients

**Fig. 6:**
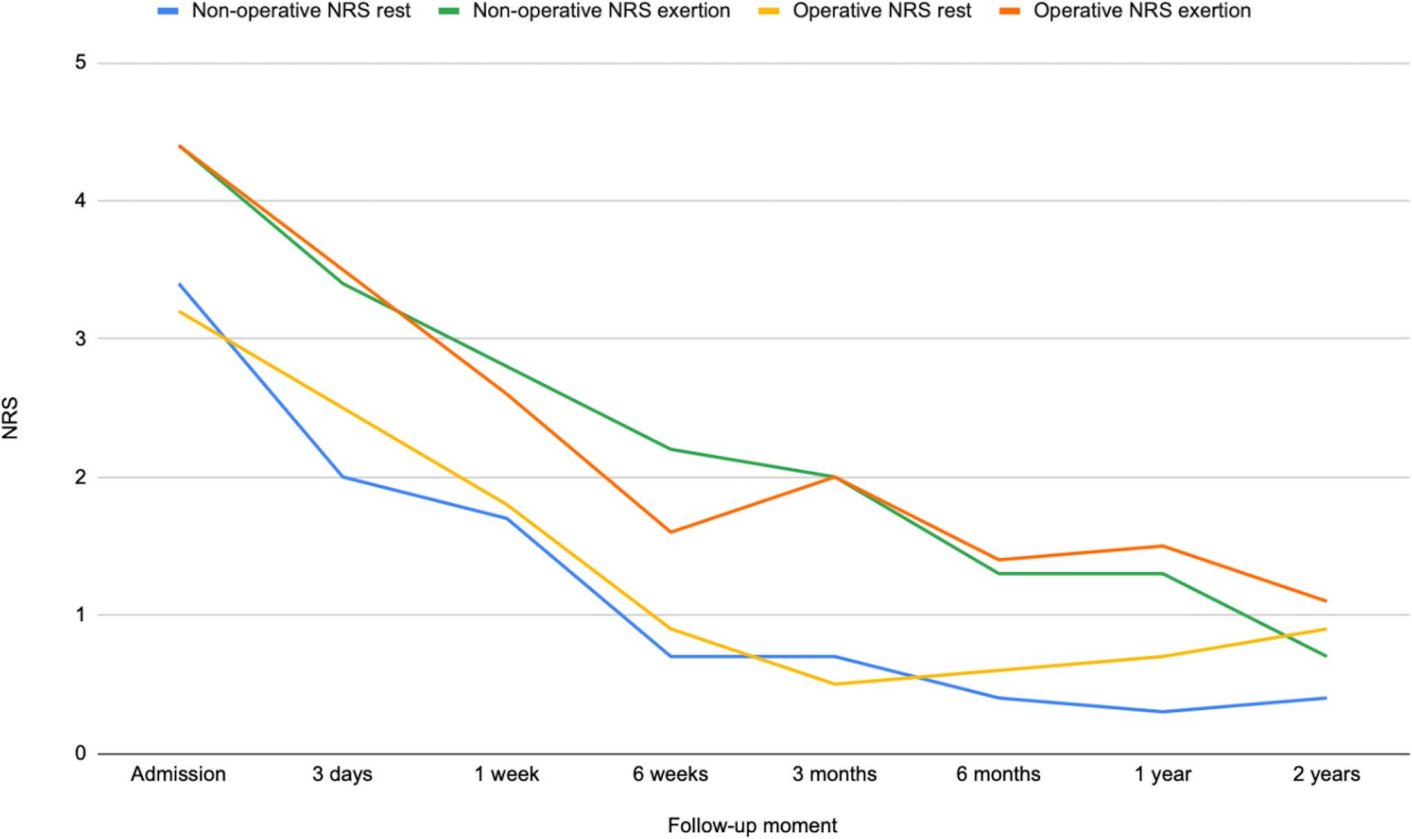
Progression of NRS pain for nonoperatively and operatively treated patients, represented by the mean NRS score at each time point at both rest and on exertion

**Fig. 7:**
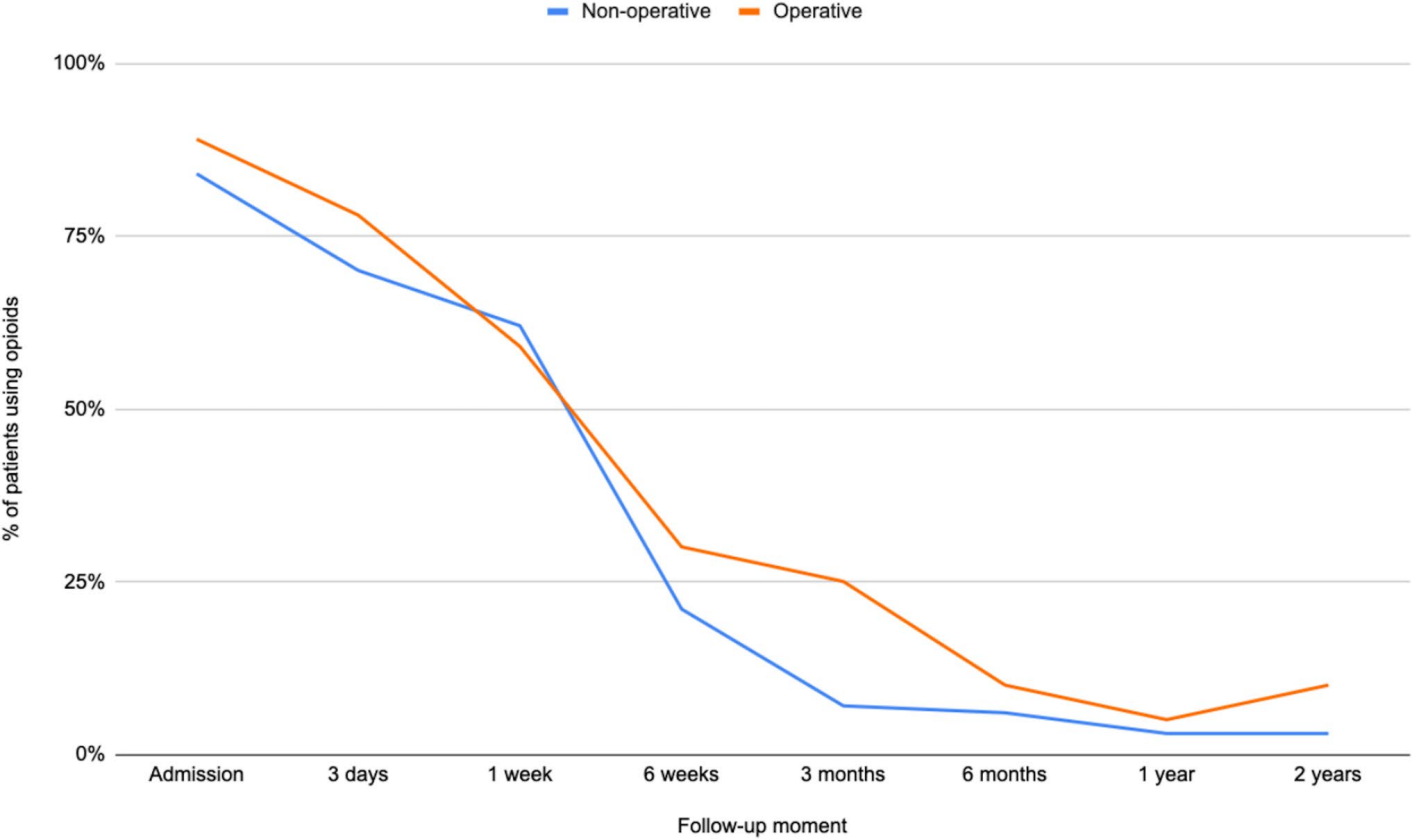
Progression of opioid use for nonoperatively and operatively treated patients, represented by the percentage of patients who use opioids at each time point

**Fig. 8:**
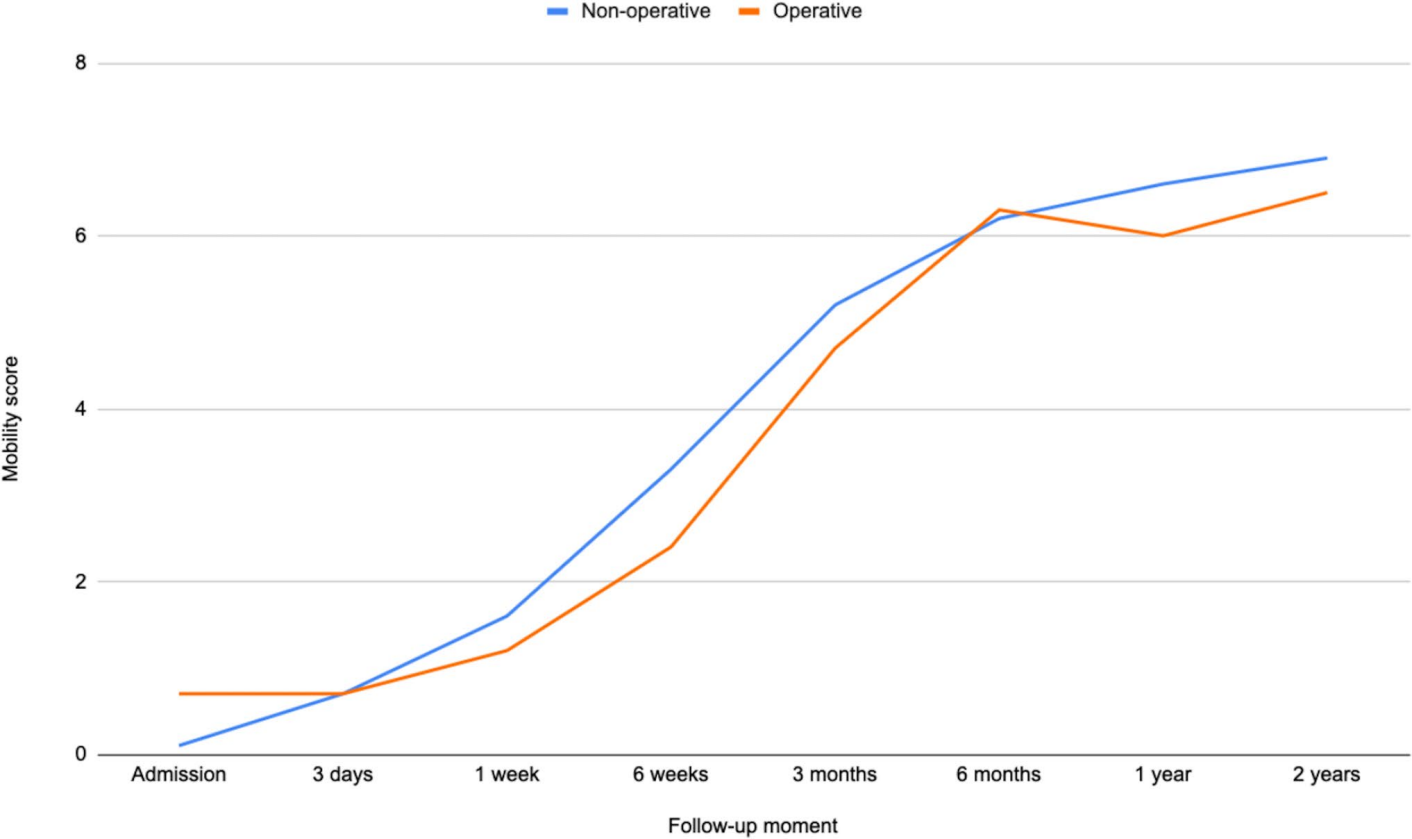
Progression of mobility for nonoperatively and operatively treated patients, represented by the mean mobility score

Results from the sub-analyses of patients who were treated nonoperatively and operatively can be viewed in Figs. 6, 7, and 8. See supplementary data for a comparison of patient characteristics, and a table with outcomes for each group. For both groups, pain and opioid use were highest, and mobility was lowest upon admission. All outcomes gradually improved throughout the recovery process. Few patients from either group experienced pain, used opioids or were not able to walk independently after two years.

## Discussion

There is a lack of data regarding the course of recovery in patients with LC pelvic ring injuries. Therefore, longitudinal data regarding pain, pain medication use, and mobility were prospectively recorded in a level-1 trauma center from the time of admission until the two-year follow-up. This allowed us to gain insight into the patient’s perception of pain, consumption of pain medication and mobility after an LC injury to better understand the course of recovery of this injury. Ninety-seven patients with an LC injury were included in the study. The outcomes were collected at eight time points to provide a comprehensive view of both the short-term and long-term course of pain recovery and mobility for this patient group. This study indicates that pain levels, both at rest and during exertion, were highest upon admission but decreased rapidly within the first six weeks. Opioid usage declined most rapidly between six weeks and three months, with few patients continuing opioid use beyond three months. Mobility in terms of walking ability rapidly improved between six weeks and three months post-injury, with the majority of patients achieving mobilization, either with the assistance of a walking aid or walking independently by the three-month mark. Following two years of recovery, almost no patients experienced pain, used opioids or were having difficulties with walking.

This study contributes value to the previous literature because it is the first longitudinal prospective study investigating pain perception, opioid use and walking ability after LC injuries. Furthermore, the subgroup analysis of nonoperatively and operatively treated patients provides valuable insight into the recovery process after the specific treatments. A prospective study by Banierink et al. [3] investigated the long-term physical functioning and quality of life after a pelvic ring injury; however, they did not provide insights about the pain, opioid use and mobility that can be expected. The study by Hagen et al. [6] did address pain, narcotic use and mobilization in patients with LC injuries, however, that study was performed retrospectively, using only three short-term time points to compare the outcomes during the initial hospital stay of patients treated conservatively and operatively. Another retrospective study by Höch et al. used pain perception as an outcome for comparing treatment methods in LC injury patients, however, only one follow-up moment (at least one year following the injury) was recorded for each patient [15].

The results from the current study can be used to inform patients and physicians about what they can expect in the first two years following an LC injury. Patients can be informed that the severity of pain decreases the most in the first weeks, and for only a few patients, there can be lasting pain or discomfort in the pelvis or lower back or due to neurologic pain. Furthermore, patients can expect not to need opioids three months after the LC injury. There are a few instances where opioids will still be needed due to pain in the pelvis or from other injuries sustained in the trauma. Lastly, it can be concluded that patients will likely be walking with the help of walking aids at the six-week follow-up moment and walking independently after 3–6 months, with nearly all patients walking independently after two years. Difficulties in walking ability can be due to comorbidities such as obesity or advanced age.

Some strengths and limitations of this study should be addressed. The longitudinal prospective study design allows there to be no recall biases for the pain perceived, opioids usage, and level of walking ability. Another strength is that the data were collected by surgeons or specialized nurses in an outpatient clinic setting. Moreover, the frequency of data collection is a noteworthy strength. The eight follow-up moments offer an extensive overview of the most critical phases during the initial two years of recovery. A limitation of the study that needs to be considered is missing data. Not all patients attended all follow-up appointments, and recording the measurements was not always feasible. At each follow-up moment, data from different patients were missing, resulting in a slightly different patient group at each follow-up time point. Moreover, the sample size was too small to run valuable subgroup analyses regarding specific injury types (LC1, LC2, LC3), as most of the study population consisted of LC1 injuries. Lastly, another limitation may be that only 40% of patients had an isolated pelvic ring injury. Thirteen percent of patients also endured a lower extremity injury during the initial trauma, which is important to consider as it could impact the patient’s ability to mobilize and their opioid usage. On the other hand, this sample size was representative of what is presented at a level-1 trauma center and is thus generalizable.

## Conclusions

This longitudinal prospective cohort study provides insights into important aspects of the course of recovery of patients with LC pelvic ring injuries by investigating pain perception, opioid use and mobility in the first two years following the injury. It can be concluded that the pain experienced rapidly decreases within the first six weeks of recovery. At the two-year follow-up, most patients will not experience any pain. Most patients will not need opioids after three months of recovery. Furthermore, most patients will be walking with the help of a walking aid after six weeks, with almost everyone walking independently at the two-year follow-up moment.

## Supplementary Information

Below is the link to the electronic supplementary material.

Supplementary file1 (DOCX 17 KB)
