# Supplementary material for: Pain perception, opioid consumption and mobility following lateral compression pelvic ring injuries: a two-year prospective cohort study
Source: Eur J Orthop Surg Traumatol. 2025 Jan 11;35(1):55. doi: 10.1007/s00590-024-04128-w (PMC11724773; doi:10.1007/s00590-024-04128-w)
Supplement: Supplementary file 1 — Supplementary file1 (DOCX 17 KB) [file 590_2024_4128_MOESM1_ESM.docx]

**Supplementary data**

**Comparison of patient and injury characteristics in non-operatively and operatively treated patients**

| **Patient characteristics** | **Non-operatively treated patients (n=74)** | **Operatively treated patients (n=23)** | **Difference, p-value** |
| --- | --- | --- | --- |
| Female, n (%) | 46 (62) | 5 (22) | <0.001 |
| Age at the time of injury, mean (SD) | 58 (20) | 54 (15) | 0.09 |
| Age>65, n (%) | 35 (47%) | 6 (13%) | 0.06 |
| Injury type, n (%) |  |  | 0.52 |
| Lateral compression 1 | 56 (76%) | 15 (65%) |  |
| Lateral compression 2 | 10 (14%) | 4 (17%) |  |
| Lateral compression 3 | 8 (11%) | 4 (17%) |  |
| High-energy trauma*, n (%) | 47 (64%) | 18 (78%) | 0.17 |
| Isolated pelvic ring injury, n (%) | 28 (38%) | 12 (52%) | 0.16 |
| Associated lower extremity injuries, n (%) | 10 (14%) | 3 (13%) | 0.63 |
| Length of hospital in days, mean (SD) | 9 (9) | 13 (8) | 0.48 |
| Discharged to care facility, n (%) | 24 (32%) | 9 (38%) | 0.19 |
| Discharged home, n (%) | 50 (68%) | 14 (61%) | 0.55 |

*Pain perception, opioid use and mobility in non-operatively treated patients (n=74)*

|  | **NRS rest,**  **mean (SD)** | **NRS exertion,**  **mean (SD)** | **Opioids,**  **n (%)** | **Mobility*,**  **mean (SD)** |
| --- | --- | --- | --- | --- |
| **Admission** | 3.4 (2.6) | 4.4 (2.8) | 59 (84%) | 0.1 (0.5) |
| **3 days** | 2.0 (1.9) | 3.4 (2.2) | 49 (70%) | 0.7 (0.9) |
| **1 week** | 1.7 (1.8) | 2.8 (2.1) | 42 (62%) | 1.6 (1.3) |
| **6 weeks** | 0.7 (1.5) | 2.2 (2.2) | 14 (21%) | 3.3 (2.4) |
| **3 months** | 0.7 (1.5) | 2.0 (2.6) | 5 (7%) | 5.2 (2.2) |
| **6 months** | 0.4 (1.2) | 1.3 (2.3) | 4 (6%) | 6.2 (1.9) |
| **1 year** | 0.3 (1.2) | 1.3 (2.5) | 2 (3%) | 6.6 (1.3) |
| **2 years** | 0.4 (1.2) | 0.7 (1.9) | 2 (3%) | 6.9 (0.7) |

* Walking ability was assessed on a mobility score of 0-7, with 0 indicating that the patient is bedridden and 7 indicating that the patient does not have impairments in walking

*Pain perception, opioid use and mobility in operatively treated patients (n=23)*

|  | **NRS rest,**  **mean (SD)** | **NRS exertion,**  **mean (SD)** | **Opioids,**  **n (%)** | **Mobility*,**  **mean (SD)** |
| --- | --- | --- | --- | --- |
| **Admission** | 3.2 (2.8) | 4.4 (2.7) | 16 (89%) | 0.7 (1.6) |
| **3 days** | 2.5 (1.9) | 3.5 (2.7) | 14 (78%) | 0.7 (1.5) |
| **1 week** | 1.8 (1.8) | 2.6 (1.9) | 13 (59%) | 1.2 (0.9) |
| **6 weeks** | 0.9 (1.9) | 1.6 (1.5) | 6 (30%) | 2.4 (1.7) |
| **3 months** | 0.5 (1.1) | 2.0 (1.9) | 5 (25%) | 4.7 (2.3) |
| **6 months** | 0.6 (1.9) | 1.4 (2.4) | 3 (10%) | 6.3 (1.8) |
| **1 year** | 0.7 (1.8) | 1.5 (3.1) | 1 (5%) | 6.0 (1.8) |
| **2 years** | 0.9 (2.3) | 1.1 (2.5) | 2 (10%) | 6.5 (0.3) |

* Walking ability was assessed on a mobility score of 0-7, with 0 indicating that the patient is bedridden and 7 indicating that the patient does not have impairments in walking
